# Changes in body weight and cardiovascular risk factors in a Chinese population with type 2 diabetes mellitus: a longitudinal study

**DOI:** 10.3389/fendo.2023.1112855

**Published:** 2023-04-12

**Authors:** Yun-Yi Li, Yu-Meng Yang, Sufen Zhu, Hui Cheng, Jose Hernandez, Wenyong Huang, Harry H. X. Wang, Yu Ting Li

**Affiliations:** ^1^ School of Public Health, Sun Yat-Sen University, Guangzhou, China; ^2^ State Key Laboratory of Ophthalmology, Zhongshan Ophthalmic Center, Sun Yat-Sen University, Guangzhou, China; ^3^ Nuffield Department of Primary Care Health Sciences, University of Oxford, Oxford, United Kingdom; ^4^ Faculty of Medicine and Health, EDU, Digital Education Holdings Ltd., Kalkara, Malta; ^5^ Green Templeton College, University of Oxford, Oxford, United Kingdom; ^6^ Guangdong Provincial Key Laboratory of Ophthalmology and Visual Science, Zhongshan Ophthalmic Center, Sun Yat-Sen University, Guangzhou, China; ^7^ JC School of Public Health and Primary Care, Faculty of Medicine, The Chinese University of Hong Kong, Hong Kong, Hong Kong SAR, China; ^8^ Usher Institute, Deanery of Molecular, Genetic & Population Health Sciences, The University of Edinburgh, Scotland, United Kingdom

**Keywords:** weight changes, cardiovascular risk factors, type 2 diabetes mellitus, primary care, general practice, community medicine

## Abstract

**Introduction:**

The primary care management of blood glucose, blood pressure, lipid profiles, and body weight is important among patients with type 2 diabetes mellitus (T2DM) to prevent disease progression. Information on how weight changes would improve or deteriorate cardiovascular (CV) risk factors is warranted for making primary care recommendations. We aimed to investigate the changes in body weight and CV risk factors and to analyse their association in a Chinese population with T2DM.

**Methods:**

We retrieved longitudinal data between 2020 and 2021 from 1,758 adult primary care patients enrolled in a diabetic retinopathy (DR) screening programme. Linear associations of changes in body weight with CV risk factors were explored. Multivariable logistic regression analysis was performed to examine associations between different weight change categories and the worsening of CV risk factors.

**Results:**

The mean age of all the participants was 63.71 years, and over half of participants were females. During a one-year follow-up period, 24.7% of patients had a weight loss of ≥3%, while 22.2% of patients had a weight gain of ≥3%. Patients who had a weight loss of ≥3% were more likely to prevent the worsening of haemoglobin A1c (HbA1c) and triglycerides, while those who had a weight gain of ≥3% tended to have worsened HbA1c, lipid profiles, and blood pressure.

**Conclusion:**

Results from this real-world investigation suggested the concurrent need for weight loss intervention among patients who are overweight or obese and weight gain prevention among patients whose body weight falls within the normal range in the context of community-based diabetes management.

## 1 Introduction

The global prevalence of diabetes was estimated at 9.3% in 2019, and it is predicted that this number will rise to 10.2% (578 million people) by 2030, and 10.9% (700 million people) by 2045 (1). Adults with type 2 diabetes mellitus (T2DM) are at high risk of cardiovascular (CV) disease, which represents the most common complication and the leading cause of mortality in people with T2DM (2, 3). This has contributed substantially to treatment costs for T2DM at both individual and population levels globally (4). Elevated blood pressure (BP), blood glucose, and lipid levels are all important physiological and biochemical risk factors closely associated with the onset of CV disease (5). Obesity is a pathological condition that plays a central role in the pathophysiology of T2DM while aggregating several CV risk factors (6–8). Weight gain has been shown to be associated with the worsening of CV risk factors and increased risk of metabolic syndrome (9, 10), yet obesity management is still challenging particularly in low- and middle-income countries (11).

Current estimates suggest that nearly 130 million people are living with diabetes in China (12), which account for nearly one-fourth of patients with diabetes worldwide. Compared to western countries where the majority of patients with diagnosed diabetes are overweight or obese (5, 13), a large proportion (43%) of patients with T2DM in China are of normal weight with a body mass index (BMI) falling within the range of 18.5 kg/m^2^ to 23.9 kg/m^2^ (14). However, previous studies on the relationship between changes in weight and a set of CV risk factors including BP, blood glucose and lipid levels were mainly conducted in the overweight or obese population, with less attention paid to people with normal weight (15–18). Further investigations are warranted to understand how weight changes may impact CV risk factors in the Chinese population with T2DM.

Much evidence from randomised controlled trials indicates that lifestyle interventions on weight management could significantly improve CV risk factors in T2DM patients (16, 19, 20). However, trials conducted in clinical settings are often difficult to reflect the exposure-outcome relationships in real-world settings (21). The significant effect achieved in clinical trials may not necessarily translate into sustainable lifestyle modification in daily practice and community settings (22, 23). Given the widespread difficulties in weight loss in diabetes management, information on how weight changes would improve or deteriorate CV risk factors is essential for making tailored primary care recommendations to successfully prevent the progression of T2DM (24).

The main objective of this study was to assess the longitudinal changes in body weight and CV risk factors in a primary care population with T2DM. The study also aimed to address the research question of whether there is a significant association between different weight change categories and changes in haemoglobin A1c (HbA1c), BP, and lipid profiles in the study population.

## 2 Materials and methods

### 2.1 Study design

This was a longitudinal, observational study conducted in Guangdong province, southern China between September 2020 and December 2021. The study was part of a larger project on screening for diabetic retinopathy (DR) in collaboration with the Guangzhou Diabetic Eye Study Group at the Zhongshan Ophthalmic Center, Sun Yat-Sen University. The anthropometric and clinical parameters including weight, height, BP, HbA1c, and lipid profiles were measured annually from September 2020 to January 2021, and from September 2021 to December 2021, respectively. The study period did not cover the Chinese New Year to minimise the possibility of acute diet change.

### 2.2 Setting and data source

The study participants were patients with diabetes who were drawn from community and township health centres. These community-based primary care facilities offer a comprehensive package of diabetes management care that is integrated as part of the free-of-charge, national basic public health service delivery (25, 26). Routine diabetic care includes periodic blood glucose tests, regular follow-up exams, and tailored advice on medicine use, diet modification, and physical exercise. An interviewer-administered questionnaire was used to collect information on socio-demographics (i.e., age, sex, education level, place of residence, marital status, living relationships, and household income), lifestyle behaviours, medical history, and drug use. The anthropometrics (i.e., height and weight) and BP parameters were measured by clinical staff. The HbA1c and lipid panel testing was performed in centralised clinical laboratory. The check-up data were retrieved electronically from the computerised records.

### 2.3 Participants

The target subjects in the study were primary care patients with clinically-diagnosed T2DM. Diabetes was diagnosed as a fasting plasma glucose level ≥7.0 mmol/L or HbA1c ≥6.5% (27). The presence of T2DM was assessed by the attending primary care physician according to the Chinese Diabetes Society guideline and the World Health Organization (WHO) recommendation (27, 28). A total of 1,795 patients with T2DM participated in both the 2020 and 2021 waves of DR screening. We excluded patients who did not have body weight measured in either wave (n=22). Patients with weight change below *P_0.5_* or above *P_99.5_* (n=15) were also excluded to take into account the possible measurement error while minimising the impact of excess weight loss or weight gain on the change of CV risk factors. This yielded a total of 1,758 patients with T2DM included in the final analysis.

### 2.4 Study variables and measurements

Self-reported information on age, sex, residence place, education level, living relationships, marital status, household income, smoking status, drinking status, duration of diabetes, medical history (e.g., hypertension, heart disease, and stroke), and current use of antihypertensive drugs and glucose-lowering agents was collected. The anthropometric parameters were measured by medical staff following a standardised protocol. Weight was measured with light clothing and without shoes by a calibrated weighing scale. Height was measured using a wall-mounted stadiometer with the position of the body being straight against the wall. The BMI was calculated as weight in kilograms divided by squared height in meters (kg/m^2^). BP was measured by routinely validated automatic sphygmomanometers at a seated position after at least 5 minutes of rest, and the arm with the higher BP values was used. The average of two BP readings, 1-2 min apart, was recorded. A fasting venous blood sample was collected. Plasma cholesterol, triglycerides, and HbA1c were measured using an automated, clinical chemistry analyzer (TBA-120FR, Toshiba, Japan) with coefficients of variation in compliance with the laboratory measurement standard.

### 2.5 Definitions

Weight gain was defined as an increase of 3% and above in body weight during 2020-2021, while weight loss was defined as a decrease of 3% and more in body weight during the same study period. A change in body weight of at least 3% has been considered clinically meaningful given that it is unlikely caused by measurement error or normal weight fluctuations (29–31). Worsening of HbA1c, triglycerides, total cholesterol, low-density lipoprotein (LDL) cholesterol, systolic BP, and diastolic BP were defined as increased values in 2021 compared with that in 2020, except for high-density lipoprotein (HDL) cholesterol which was defined as decrease values in 2021 compared with that in 2020.

### 2.6 Statistical analysis

Data entry was performed by two trained research assistants with double verification using EpiData 3.1 (Denmark). Descriptive statistics were used to describe the demographics, lifestyle behaviours, medical history, and clinical parameters of patients according to different weight change categories. Between-group differences were assessed by independent *t*-tests or chi-square tests, where appropriate. Pearson correlation between weight change and CV risk factor change was determined. The proportion of patients with worsening CV risk factors were plotted against different weight change categories, and tests for trend were conducted. Multivariable logistic regression analysis was performed to explore the association with a 95% confidence interval (95%CI) between different weight change categories and worsening of CV risk factors after adjusting for age, sex, baseline BMI, education level, residence place, marital status, living relationships, household income, smoking status, alcohol drinking, duration of diabetes, antihypertensive medication use, oral hypoglycaemic drugs use, insulin use, and presence of CV comorbidities. Data analysis was conducted using Stata 14 (StataCorp, TX). A *p*-value <0.05 was considered statistically significant.

### 2.7 Ethics consideration

All participants provided written informed consent. Data anonymisation was performed by removing all patient identifiers from the dataset prior to data analysis. Ethics approval was granted by the Zhongshan Ophthalmic Center Medical Ethics Committee (Ref: 2017KYPJ094) at Sun Yat-Sen University following the Declaration of Helsinki 2013.

## 3 Results

### 3.1 Characteristics of study participants

A total of 1,758 patients with T2DM who met the eligibility criteria were included in the study. The mean age and baseline BMI of all participants was 63.71 years (standard deviation [SD]: 9.39) and 24.36 kg/m^2^ (SD: 3.36), respectively. More than half of the participants (56.3%) were females. Almost one-fourth (24.7%) of patients had a weight loss of ≥3%, and their mean baseline BMI was 24.63 kg/m^2^. Slightly above one-fifth (22.2%) of patients had a weight gain of ≥3%, and their mean baseline BMI was 23.39 kg/m^2^. The weight gain group had the highest proportion of patients with formal education and those who were rural residents **(** **Table 1** **)**. A higher proportion of patients who had concurrent hypertension and antihypertensive drug use was observed in the weight loss group **(** **Table 2** **)**. There were no significant differences in the distribution of age, sex, marital status, living relationships, smoking status, alcohol drinking, household income, duration of diabetes, presence of CV comorbidities, and glucose-lowering medication use across the three groups.

**Table 1:** Baseline characteristics of study participants by different weight change categories.

Variables	All patients (N =1,758)	Weight loss (n=434)	Weight stability (n=933)	Weight gain (n=391)	*p*-value
Age, years	63.71 (9.39)	63.69 (9.52)	64.05 (9.32)	62.90 (9.40)	0.123
Sex					
Male	768 (43.7%)	183 (42.2%)	415 (44.5%)	170 (43.5%)	0.721
Female	990 (56.3%)	251 (57.8%)	518 (55.5%)	221 (56.5%)	
Education level					
No formal education	575 (32.7%)	171 (39.4%)	288 (30.9%)	116 (29.7%)	0.003
Primary school and above	1,183 (67.3%)	263 (60.6%)	645 (69.1%)	275 (70.3%)	
Place of residence					
Rural	1,142 (65.0%)	294 (67.7%)	574 (61.5%)	274 (70.1%)	0.004
Urban	616 (35.0%)	140 (32.3%)	359 (38.5%)	117 (29.9%)	
Marital status					
Married	1,441 (82.0%)	357 (82.3%)	764 (81.9%)	320 (81.8%)	0.984
Others	317 (18.0%)	77 (17.7%)	169 (18.1%)	71 (18.2%)	
Living relationships					
Living alone	220 (12.5%)	41 (9.4%)	127 (13.6%)	52 (13.3%)	0.083
Living with a partner	1,538 (87.5%)	393 (90.6%)	806 (86.4%)	339 (86.7%)	
Smoking status					
Current smoking	300 (17.1%)	78 (18.0%)	152 (16.3%)	70 (17.9%)	0.657
Others	1,458 (82.9%)	356 (82.0%)	781 (83.7%)	321 (82.1%)	
Alcohol drinking					
Regular drinking	193 (11.0%)	37 (8.5%)	115 (12.3%)	41 (10.5%)	0.105
Others	1,565 (89.0%)	397 (91.5%)	818 (87.7%)	350 (89.5%)	
Household income, CNY					
<3,000 per month	1,293 (73.8%)	332 (76.9%)	672 (72.1%)	289 (74.3%)	0.173
≥3,000 per month	460 (26.2%)	100 (23.1%)	260 (27.9%)	100 (25.7%)	
Baseline BMI, kg/m^2^	24.36 (3.36)	24.63 (3.29)	24.64 (3.41)	23.39 (3.16)	<0.001

BMI, body mass index. Weight gain was defined as an increase of ≥3% in body weight, and weight loss was defined as a decrease of ≥3% in body weight. Weight stability was defined as having a weight change of <3% in body weight during the study period.

**Table 2:** Medical history and use of medications by different weight change categories.

Variables	All patients (N =1,758)	Weight loss (n=434)	Weight stability (n=933)	Weight gain (n=391)	*p*-value
Duration of T2DM					
<10 years	1,288 (73.3%)	319 (73.5%)	680 (72.9%)	289 (74.1%)	0.896
≥10 years	469 (26.7%)	115 (26.5%)	253 (27.1%)	101 (25.9%)	
Presence of hypertension					
Yes	725 (41.2%)	192 (44.2%)	397 (42.6%)	136 (34.8%)	0.011
No	1,033 (58.8%)	242 (55.8%)	536 (57.4%)	255 (65.2%)	
Presence of CV disease					
Yes	244 (13.9%)	61 (14.1%)	125 (13.4%)	58 (14.8%)	0.783
No	1,514 (86.1%)	373 (85.9%)	808 (86.6%)	333 (85.2%)	
Use of antihypertensive drugs					
Yes	618 (35.2%)	163 (37.6%)	339 (36.3%)	116 (29.7%)	0.033
No	1,140 (64.8%)	271 (62.4%)	594 (63.7%)	275 (70.3%)	
Use of oral hypoglycaemic drugs					
Yes	1,304 (74.2%)	329 (75.8%)	692 (74.2%)	283 (72.4%)	0.532
No	454 (25.8%)	105 (24.2%)	241 (25.8%)	108 (27.6%)	
Use of insulin					
Yes	179 (10.2%)	34 (7.8%)	107 (11.5%)	38 (9.7%)	0.111
No	1,579 (89.8%)	400 (92.2%)	826 (88.5%)	353 (90.3%)	

T2DM, type 2 diabetes mellitus; CV, cardiovascular. Weight gain was defined as an increase of ≥3% in body weight, and weight loss was defined as a decrease of ≥3% in body weight. Weight stability was defined as having a weight change of <3% in body weight during the study period.

### 3.2 Changes in body weight and CV risk factors

Haemoglobin A1c, HDL cholesterol, and triglycerides decreased significantly between 2020 and 2021, while LDL cholesterol increased during the study period. We did not observe significant changes in body weight, total cholesterol, systolic BP, and diastolic BP over time. In addition, we observed significant correlations of weight change with a set of changes in HbA1c (Pearson correlation [*r*]=0.096, *p*<0.001), LDL cholesterol (*r*=0.070, *p*=0.007), triglycerides (*r*=0.133, *p*<0.001), total cholesterol (*r*=0.070, *p*=0.007), systolic BP (*r*=0.136, *p*<0.001), and diastolic BP (*r*=0.083, *p*<0.001). There was also no significant correlation between weight change and change in HDL cholesterol **(** **Table 3** **)**.

**Table 3:** Changes in CV risk factors and their correlations with weight change.

	Baseline		Changes during 2020-2021		Correlation with weight change
	N	Mean ± SD	N	Mean (95%CI)	
Weight, kg	1,758	59.79 ± 10.44	1,758	-0.02 (-0.15, 0.11)	
HbA1c, %	1,658	7.98 ± 2.39	1,503	-0.95 (-1.07, -0.84)†	0.096†
LDL cholesterol, mmol/L	1,659	2.24 ± 0.78	1,505	0.26 (0.22, 0.30)†	0.070*
HDL cholesterol, mmol/L	1,662	1.41 ± 0.65	1,508	-0.07 (-0.11, -0.03)†	-0.023
Triglycerides, mmol/L	1,662	2.19 ± 1.78	1,507	-0.09 (-0.17, -0.004)*	0.133†
Total cholesterol, mmol/L	1,662	5.28 ± 1.39	1,505	0.04 (-0.02, 0.11)	0.070*
Systolic BP, mmHg	1,752	141.46 ± 19.24	1,745	0.76 (-0.14, 1.66)	0.136†
Diastolic BP, mmHg	1,752	84.14 ± 11.18	1,743	-0.12 (-0.69, 0.45)	0.083†

* p<0.05, † p<0.001.

HbA1c, haemoglobin A1c; LDL, low-density lipoprotein; HDL, high-density lipoprotein; BP, blood pressure.

### 3.3 Worsening of CV risk factors across different weight change categories

**Figure 1:**
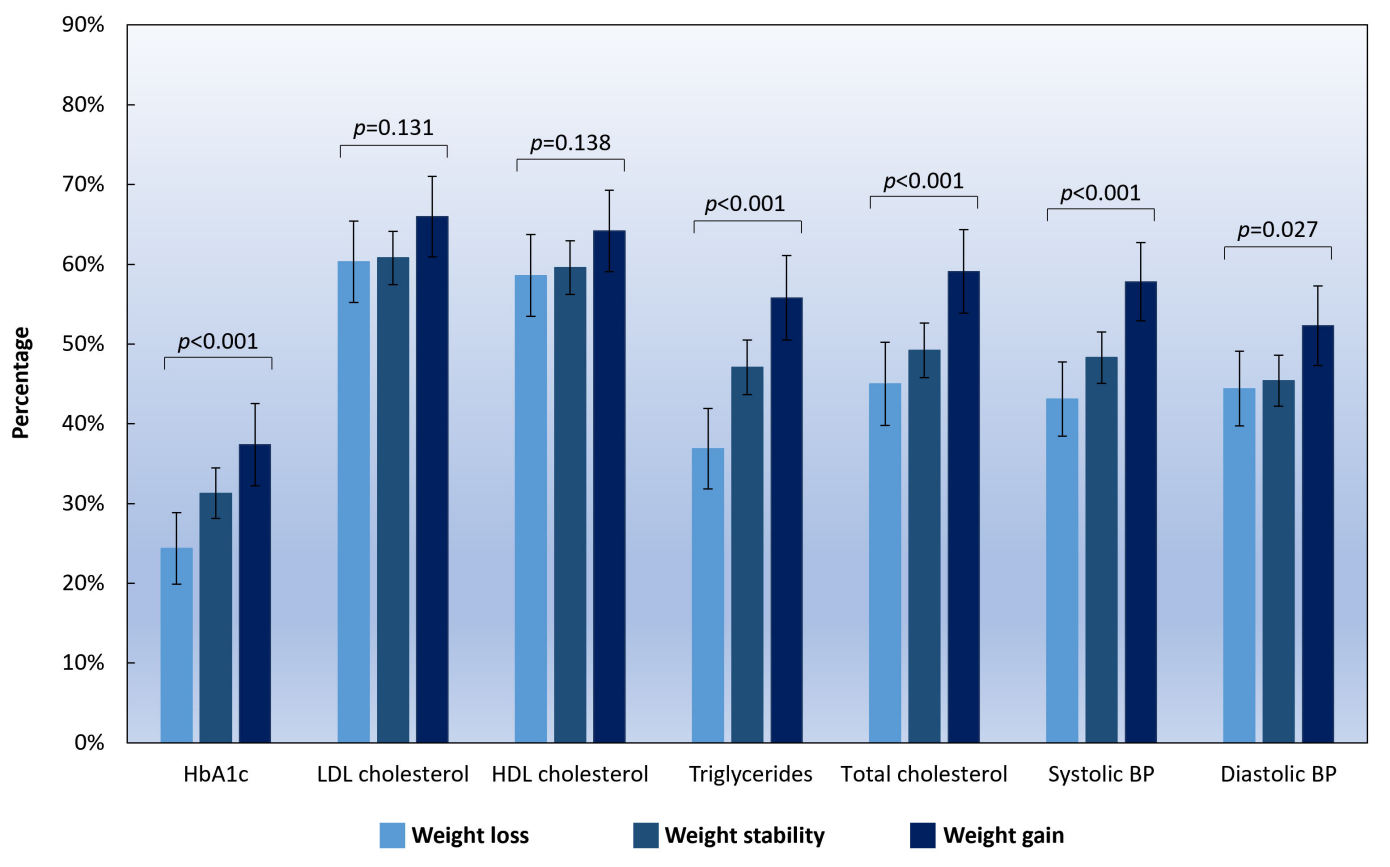
Percentage of patients who had worsening CV risk factors according to different weight change categories. Note: HbA1c, haemoglobin A1c; LDL, low-density lipoprotein; HDL, high-density lipoprotein; BP, blood pressure. Weight gain was defined as an increase of ≥3% in body weight, and weight loss was defined as a decrease of ≥3% in body weight. Weight stability was defined as having a weight change of <3% in body weight during the study period. Error bars indicate 95% confidence intervals.

The highest proportion of patients with worsening CV risk factors was found in the weight gain group, where 37.4% of patients had increased HbA1c, 66.0% had increased LDL cholesterol, 64.2% had decreased HDL cholesterol, 55.8% had increased triglycerides, 59.1% had increased total cholesterol, 57.8% had increased systolic BP, and 52.3% had increased diastolic BP. An increasing trend in the proportion of patients with the worsening of HbA1c (*p*<0.001), triglycerides (*p*<0.001), total cholesterol (*p*<0.001), systolic BP (*p*<0.001), and diastolic BP (*p*=0.027) was observed across the three groups from weight loss to weight gain **(** **Figure 1** **)**.

### 3.4 Associations between different weight categories and worsening of CV risk factors

**Figure 2:**
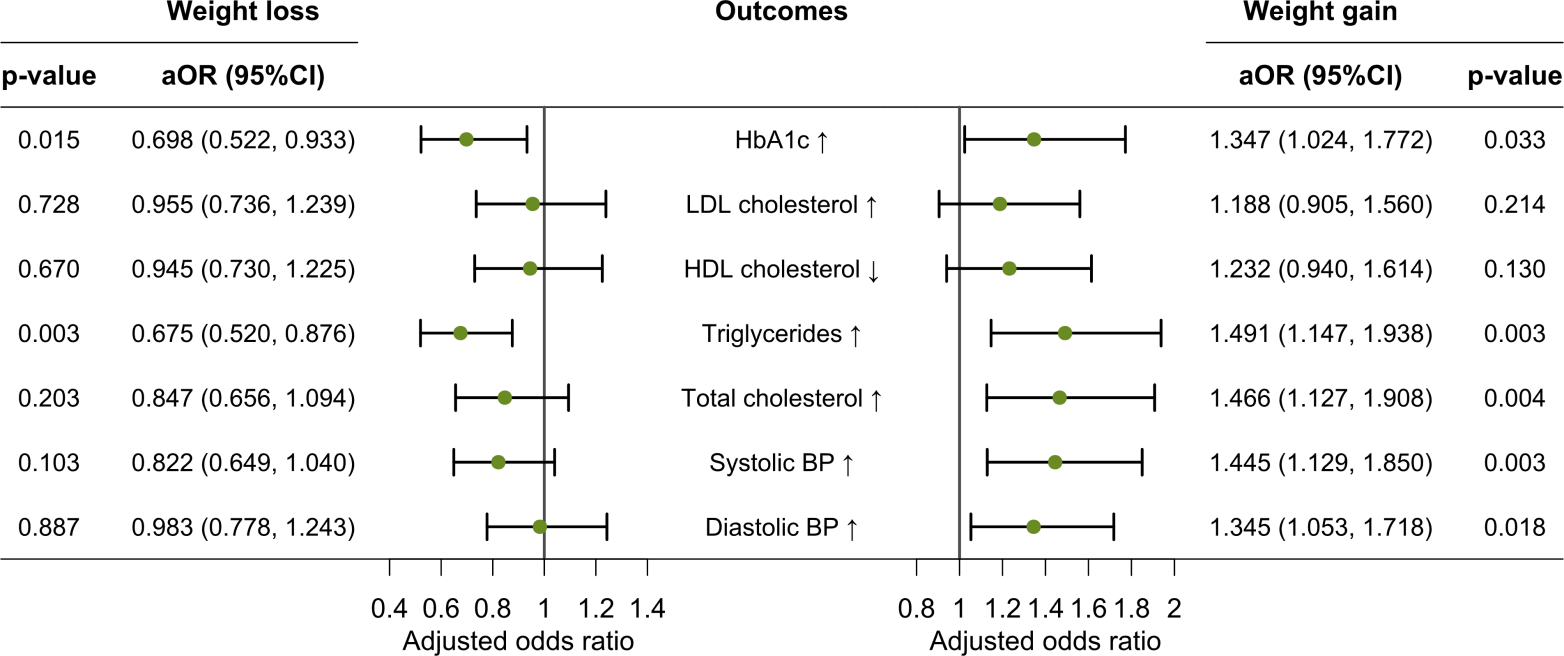
Multivariable logistic regression analysis of associations between weight loss/gain and worsening of CV risk factors. Note: aOR, adjusted odds ratio; HbA1c, haemoglobin A1c; LDL, low-density lipoprotein; HDL, high-density lipoprotein; BP, blood pressure. Weight gain was defined as an increase of ≥3% in body weight, and weight loss was defined as a decrease of ≥3% in body weight during the study period. Error bars indicate 95% confidence intervals.

The multivariable logistic regression analysis showed that having a weight loss of ≥3% was negatively associated with increased HbA1c (adjusted odds ratio [aOR]=0.698, 95%CI: 0.522-0.933, *p*=0.015) and increased triglycerides (aOR=0.675, 0.520-0.876, *p*=0.003) after adjusting for age, sex, baseline BMI, education level, residence place, marital status, living relationships, household income, smoking status, alcohol drinking, duration of diabetes, antihypertensive medication use, oral hypoglycaemic drugs use, insulin use, and presence of CV comorbidities. Meanwhile, having a weight gain of ≥3% was positively associated with increased HbA1c (aOR=1.347, 1.024-1.772, *p*=0.033), increased triglycerides (aOR=1.491, 1.147-1.938, *p*=0.003), increased total cholesterol (aOR=1.466, 1.127-1.908, *p*=0.004), increased systolic BP (aOR=1.445, 1.129-1.850, *p*=0.003), and increased diastolic BP (aOR=1.345, 1.053-1.718, *p*=0.018) **(** **Figure 2** **)**.

## 4 Discussion

### 4.1 Main findings

We examined changes in body weight and CV risk factors during a one-year follow-up period in a Chinese primary care population with T2DM. Nearly one in four patients had a weight loss of ≥3%, while slightly above one-fifth of patients had a weight gain of ≥3%. Significant linear correlations were found between weight change and a set of changes in HbA1c, LDL cholesterol, triglycerides, total cholesterol, and BP. The multivariable logistic regression analysis demonstrated that patients who had a weight loss of ≥3% were more likely to prevent the worsening of HbA1c and triglycerides while having a weight gain of ≥3% was found to be associated with the worsening of HbA1c, triglycerides, total cholesterol, and BP.

### 4.2 Relationship with other studies

Data from the United States described a growing toll of diabetes and obesity-related CV disease despite a decline in CV mortality over the past four decades (32). Global estimates of disease burden showed that CV disease attributable to elevated BMI was the main cause of death and disability-adjusted life years, accounting for 2.7 million deaths and 66.3 million disability-adjusted life years (33). Weight gain has been shown to induce early onset and accumulation of vascular risk factors, which are closely linked to cardiometabolic abnormalities such as atherosclerosis (34). Earlier findings from a large population-based cohort study in China suggested that the CV risk started to increase with mildly elevated body weight (23-25 kg/m^2^) (35) – a level that is considered normal weight according to the WHO criteria (36). A cohort study conducted in the American population reported that people with weight-gain patterns were more prone to have above-goal HbA1c and BP than their counterparts with weight loss (15). This was similarly observed in our study in which having a weight gain of 3% and above was significantly associated with worsening of CV risk factors including HbA1c, triglycerides, total cholesterol, and both systolic and diastolic BP. The results were in line with our expectations and the existing literature which suggested that a progression from non-obese to becoming obese was accompanied by an increment in predicted risk for CV disease in the Japanese community residents (9). It is possible that low-grade inflammation and dysregulation of the endocrine and immune milieu in the adipose tissue could lead to abnormal production of adipokines and inflammatory molecules (37). Previous evidence also suggested a link between weight gain and increased risk of vascular dysfunction in patients with T2DM (38), which implies that physicians should move beyond a simple focus on glycaemic control and takes into account the importance of weight management in diabetes care.

Previous epidemiological studies have established that the rising prevalence of overweight and obesity contributes to the increased incidence of diabetes and CV disease (39–41). Weight loss has been incorporated as one of the major goals of interventions in T2DM patients who are overweight or obese to prevent the development of CV disease. An observational analysis of obese patients with T2DM in the Look AHEAD study showed that weight losses of 5 to <10% were associated with significant improvements in HbA1c, BP, triglycerides, and HDL cholesterol (17). Our data showed that weight loss of ≥3% was strongly associated with improved HbA1c and triglycerides. The benefits of weight loss on other CV risk factors such as total cholesterol and BP were also observed in our study albeit not statistically significant. This may be due to the use of a more conservative threshold level of 3% instead of 5% for determining the meaningful magnitude of weight change in our study given that achieving a greater level of weight loss requires intense interventions (16), which, however, was absent in the present study. It might also be explained by the increased fitness as a result of the long-term lifestyle intervention in the Look AHEAD study, which may improve CV risk factors beyond weight loss alone and further enhance the beneficial effect (42, 43). Nevertheless, we did observe significant associations of weight change with improved HbA1c, triglycerides, total cholesterol, systolic BP, and diastolic BP among patients who had a weight gain of ≥3% in our study. This implies the consistency of evidence that relates weight change to CV risk factors.

### 4.3 Implications for research and practice

Regular monitoring of BP, lipid profiles, and body weight has been emphasised in diabetes management (44, 45). A modest weight loss has been considered beneficial to improving glycaemic control, BP, lipid profiles, and metabolic parameters in T2DM patients (16, 17). Nevertheless, there remain multiple barriers to effective and sustainable weight management. First, it tends to be more difficult to lose weight for people with diabetes compared to those free of diabetes (46). Second, adherence to long-term lifestyle advice such as diet modification and aerobic exercise appears to be complex and challenging (47, 48). A meta-analysis has revealed the difficulties in achieving a weight loss of more than 5% in most lifestyle weight-loss interventions (16). Third, certain antidiabetic agents may cause weight gain and thereby exacerbate other CV risk factors associated with T2DM (49). Our study suggested a beneficial impact of weight loss of ≥3% on CV risk factors. In the Chinese guideline on prevention and treatment of T2DM, achieving a weight loss of 3% to 5% has been considered fundamental to weight management for overweight or obese adults with T2DM (45). Previous real-world studies exhibited that in patients who were newly treated for T2DM, those with weight loss of ≥3% were more likely to achieve better glycaemic control (50). It is worth noting that the main purpose of our study was not to explore the threshold level *per se* for weight change. Instead, we are interested in understanding whether having a weight loss at a modest level that is culturally feasible in real-world settings could be associated with beneficial outcomes. Given the worsening of CV risk factors due to weight gain, regular monitoring of body weight should not be neglected in people whose BMI falls within the normal range. Maintaining optimal weight control shall necessitate appropriately designed health communication efforts (51), which could be made *via* interpersonal or mass media channels to reinforce diet, physical activity, and behavioural changes (52). Existing approaches for weight management advocate a cohesive engagement of primary care practitioners and multidisciplinary teams to overcome a variety of obstacles that hinder effective nonpharmacologic and pharmacologic treatment (53–55).

### 4.4 Strengths and weaknesses of the study

We presented primary care longitudinal data that reinforced the primacy of weight management as integral to diabetes care, while extending the existing evidence to a Chinese population with a particular focus on the changes in body weight and its association with changes in CV risk factors in the real-world setting. Unlike many other studies conducted in the west, nearly half of participants in our study had their BMI falling within the normal range at baseline. Both urban and rural participants were included to take into account the socioeconomic disparities. A broad range of information on patients’ demographics, lifestyle behaviours, medical history, current use of antihypertensive drugs and glucose-lowering agents, presence of CV comorbidities, and clinical parameters was collected. All clinical measurements including physical examination and laboratory tests were performed under routine check-up procedures with quality control. However, our study had several limitations. First, lifestyle behaviours were considered confounding factors and were measured in a simplified dichotomous manner, which precluded the evaluation of the frequency and intensity of physical exercise, and intake of dietary compositions (56, 57). It is also possible that smoking cessation could be accompanied by weight gain, and may impact the association between weight gain and changes in CV outcomes (58, 59). Second, we did not have information on how weight loss was achieved, nor whether an individual’s weight loss was intentional or secondary to disease. As indicated by earlier evidence, it is reasonable to assume a greater benefit of weight loss in patients who had the intention to lose weight (60). Third, we did not gather information on the fluctuation of weight during the period before baseline, and thus we cannot rule out the possibility that an individual was gaining or losing weight at study entry. Fourth, although the use of antihypertensive drugs, oral hypoglycaemic drugs and insulin was taken into account in the regression analysis, we were not able to determine whether there were changes either in diabetes medication (e.g., initiation of SGLT2i or GLP-1 receptor agonists) or in concomitant medications that might have affected weight or CV risk factors (e.g., initiation of or change in the dose of statin) during the course of this study. Last but not least, associations between the magnitude of weight change and subsequent CV outcomes may vary after the one-year follow-up period, which may warrant evidence from further long-term investigations with the assistance of wearable devices, digital eHealth platforms, and remote patient monitoring tools to guide clinical decisions in primary care (61).

## 5 Conclusion

Our data from a primary care population of T2DM patients demonstrated the longitudinal changes in body weight along with a set of concurrent changes in CV risk factors in the real-world setting. Patients who had a weight loss of ≥3% were more likely to prevent the worsening of HbA1c and triglycerides, while those who had a weight gain of ≥3% tended to have worsened HbA1c, lipid profiles, and blood pressure. Our results suggested the concurrent need for weight loss intervention among patients who are overweight or obese and weight gain prevention among patients whose body weight falls within the normal range in the context of community-based diabetes management.

## Data availability statement

The raw data supporting the conclusions of this article are available on reasonable request from the corresponding authors.

## Ethics statement

The studies involving human participants were reviewed and approved by the Ethics Committee at Zhongshan Ophthalmic Center, Sun Yat-Sen University. The patients/participants provided their written informed consent to participate in this study.

## Author contributions

Conceptualisation: Y-YL, HHXW, and YTL; data curation: WH and YTL; formal analysis: Y-YL; validation: Y-MY; methodology: Y-YL and HHXW; project administration: YTL; supervision: WH and HHXW; writing—original draft preparation: Y-YL, HHXW, and YTL; writing—review and editing: SZ, HC, and JH. All authors contributed to the article and approved the submitted version.

## References

[1] Saeedi P Petersohn I Salpea P Malanda B Karuranga S Unwin N. Global and regional diabetes prevalence estimates for 2019 and projections for 2030 and 2045: Results from the international diabetes federation diabetes atlas, 9^th^ edition. Diabetes Res Clin Pract (2019) 157:107843. doi: 10.1016/j.diabres.2019.107843 31518657

[2] Einarson TR Acs A Ludwig C Panton UH. Prevalence of cardiovascular disease in type 2 diabetes: A systematic literature review of scientific evidence from across the world in 2007-2017. Cardiovasc Diabetol (2018) 17(1):83. doi: 10.1186/s12933-018-0728-6 29884191 PMC5994068

[3] Zhang Y Hu G Yuan Z Chen L. Glycosylated hemoglobin in relationship to cardiovascular outcomes and death in patients with type 2 diabetes: A systematic review and meta-analysis. PloS One (2012) 7:e42551. doi: 10.1371/journal.pone.0042551 22912709 PMC3415427

[4] Einarson TR Acs A Ludwig C Panton UH. Economic burden of cardiovascular disease in type 2 diabetes: A systematic review. Value Health (2018) 21(7):881–90. doi: 10.1016/j.jval.2017.12.019 30005761

[5] Chatterjee S Khunti K Davies MJ. Type 2 diabetes. Lancet. (2017) 389:2239–51. doi: 10.1016/S0140-6736(17)30058-2 28190580

[6] Vázquez LA Rodríguez Á Salvador J Ascaso JF Petto H Reviriego J. Relationships between obesity, glycemic control, and cardiovascular risk factors: A pooled analysis of cross-sectional data from Spanish patients with type 2 diabetes in the preinsulin stage. BMC Cardiovasc Disord (2014) 14:153. doi: 10.1186/1471-2261-14-153 25361574 PMC4228158

[7] Tzotzas T Evangelou P Kiortsis DN. Obesity, weight loss and conditional cardiovascular risk factors. Obes Rev (2011) 12(5):e282–9. doi: 10.1111/j.1467-789X.2010.00807.x 21054756

[8] Kim SH. Cardiovascular risk factors and obesity in adolescents. Korean Circ J (2020) 50(8):733–5. doi: 10.4070/kcj.2020.0247 PMC7390712 32725981

[9] Honda T Ishida Y Oda M Noguchi K Chen S Sakata S. Changes in body weight and concurrent changes in cardiovascular risk profiles in community residents in Japan: the hisayama study. J Atheroscler Thromb (2022) 29(2):252–67. doi: 10.5551/jat.59394 PMC8803559 33455974

[10] Sun J Xi B Yang L Zhao M Juonala M Magnussen CG. Weight change from childhood to adulthood and cardiovascular risk factors and outcomes in adulthood: A systematic review of the literature. Obes Rev (2021) 22(3):e13138. doi: 10.1111/obr.13138 32875696

[11] Ford ND Patel SA Narayan KM. Obesity in low- and middle-income countries: Burden, drivers, and emerging challenges. Annu Rev Public Health (2017) 38:145–64. doi: 10.1146/annurev-publhealth-031816-044604 28068485

[12] Li Y Teng D Shi X Qin G Qin Y Quan H. Prevalence of diabetes recorded in mainland China using 2018 diagnostic criteria from the American diabetes association: National cross sectional study. BMJ (2020) 369:m997. doi: 10.1136/bmj.m997 32345662 PMC7186854

[13] Centers for Disease Control and Prevention (CDC). Prevalence of overweight and obesity among adults with diagnosed diabetes–united states, 1988-1994 and 1999-2002. MMWR Morb Mortal Wkly Rep (2004) 53:1066–8. 15549021

[14] Lv F Cai X Lin C Hong T Zhang X Wang Z. Sex differences in the prevalence of obesity in 800,000 Chinese adults with type 2 diabetes. Endocr Connect. (2021) 10:139–45. doi: 10.1530/EC-20-0547 PMC7983479 33543732

[15] Feldstein AC Nichols GA Smith DH Stevens VJ Bachman K Rosales AG. Weight change in diabetes and glycemic and blood pressure control. Diabetes Care (2008) 31(10):1960–5. doi: 10.2337/dc08-0426 PMC2551635 18697899

[16] Franz MJ Boucher JL Rutten-Ramos S VanWormer JJ. Lifestyle weight-loss intervention outcomes in overweight and obese adults with type 2 diabetes: A systematic review and meta-analysis of randomized clinical trials. J Acad Nutr Diet. (2015) 115(9):1447–63. doi: 10.1016/j.jand.2015.02.031 25935570

[17] Wing RR Lang W Wadden TA Safford M Knowler WC Bertoni AG. Benefits of modest weight loss in improving cardiovascular risk factors in overweight and obese individuals with type 2 diabetes. Diabetes Care (2011) 34:1481–6. doi: 10.2337/dc10-2415 PMC3120182 21593294

[18] Blonde L Pencek R MacConell L. Association among weight change, glycemic control, and markers of cardiovascular risk with exenatide once weekly: A pooled analysis of patients with type 2 diabetes. Cardiovasc Diabetol (2015) 14:12. doi: 10.1186/s12933-014-0171-2 25645567 PMC4324846

[19] Lean ME Leslie WS Barnes AC Brosnahan N Thom G McCombie L. Primary care-led weight management for remission of type 2 diabetes (DiRECT): an open-label, cluster-randomised trial. Lancet. (2018) 391(10120):541–51. doi: 10.1016/S0140-6736(17)33102-1 29221645

[20] Zomer E Gurusamy K Leach R Trimmer C Lobstein T Morris S. Interventions that cause weight loss and the impact on cardiovascular risk factors: a systematic review and meta-analysis. Obes Rev (2016) 17(10):1001–11. doi: 10.1111/obr.12433 27324830

[21] Heneghan C Goldacre B Mahtani KR. Why clinical trial outcomes fail to translate into benefits for patients. Trials. (2017) 18(1):122. doi: 10.1186/s13063-017-1870-2 28288676 PMC5348914

[22] Dunbar JA Hernan AL Janus ED Vartiainen E Laatikainen T Versace VL. Challenges of diabetes prevention in the real world: results and lessons from the Melbourne diabetes prevention study. BMJ Open Diabetes Res Care (2015) 3(1):e000131. doi: 10.1136/bmjdrc-2015-000131 PMC4597415 26464804

[23] Pomeroy J Palacios C. Translating findings from lifestyle intervention trials of cardiovascular disease and diabetes to the primary care setting. Curr Nutr Rep (2012) 1(4):215–21. doi: 10.1007/s13668-012-0024-0 PMC5319565 28232875

[24] Li Y Zhong Q Zhu S Cheng H Huang W Wang HHX. Frequency of follow-up attendance and blood glucose monitoring in type 2 diabetic patients at moderate to high cardiovascular risk: A cross-sectional study in primary care. Int J Environ Res Public Health (2022) 19(21):14175. doi: 10.3390/ijerph192114175 36361055 PMC9656430

[25] Department of Primary Health Care National Health Commission. PRC. standards for national basic public health services (2017). Available at: http://www.nhc.gov.cn/jws/s3578/201703/d20c37e23e1f4c7db7b8e25f34473e1b.shtml (Accessed 31 January 2023).

[26] Yao J Wang H Yin J Shao D Guo X Sun Q. Factors associated with the utilization of community-based diabetes management care: A cross-sectional study in Shandong province, China. BMC Health Serv Res (2020) 20(1):407. doi: 10.1186/s12913-020-05292-5 32393254 PMC7212576

[27] Weng J Ji L Jia W Lu J Zhou Z Zou D. Chinese Diabetes society. standards of care for type 2 diabetes in China. Diabetes Metab Res Rev (2016) 32:442–58. doi: 10.1002/dmrr.2827 PMC5108436 27464265

[28] World Health Organization & International Diabetes Federation. Definition and diagnosis of diabetes mellitus and intermediate hyperglycaemia: report of a WHO/IDF consultation. (Geneva, Switzerland: World Health Organization) (2006).

[29] Stevens J Truesdale KP McClain JE Cai J. The definition of weight maintenance. Int J Obes (Lond). (2006) 30:391–9. doi: 10.1038/sj.ijo.0803175 16302013

[30] Jakicic JM Otto AD Lang W Semler L Winters C Polzien K. The effect of physical activity on 18-month weight change in overweight adults. Obes (Silver Spring) (2011) 19:100–9. doi: 10.1038/oby.2010.122 PMC4427898 20539299

[31] McAdam-Marx C Bellows BK Unni S Wygant G Mukherjee J Ye X. Impact of adherence and weight loss on glycemic control in patients with type 2 diabetes: cohort analyses of integrated medical record, pharmacy claims, and patient-reported data. J Manag Care Spec Pharm (2014) 20:691–700. doi: 10.18553/jmcp.2014.20.7.691 24967522 PMC10437951

[32] Mensah GA Wei GS Sorlie PD Fine LJ Rosenberg Y Kaufmann PG. Decline in cardiovascular mortality: Possible causes and implications. Circ Res (2017) 120(2):366–80. doi: 10.1161/CIRCRESAHA.116.309115 PMC5268076 28104770

[33] GBD 2015 Obesity Collaborators Afshin A MH F MB R Sur P Estep K. Health effects of overweight and obesity in 195 countries over 25 years. N Engl J Med (2017) 377(1):13–27. doi: 10.1056/NEJMoa1614362 28604169 PMC5477817

[34] Ryder JR Xu P Inge TH Xie C Jenkins TM Hur C. Thirty-year risk of cardiovascular disease events in adolescents with severe obesity. Obes (Silver Spring) (2020) 28(3):616–23. doi: 10.1002/oby.22725 PMC7045971 32090509

[35] Zhu Y Zheng R Hu C Qin G Wang B Wang T. Association of early adulthood weight and subsequent weight change with cardiovascular diseases: Findings from REACTION study. Int J Cardiol (2021) 332:209–15. doi: 10.1016/j.ijcard.2021.02.086 33667580

[36] WHO Global InfoBase team. The SuRF report 2. In: Surveillance of chronic disease risk factors: Country-level data and comparable estimates. (Geneva, Switzerland: World Health Organization) (2005).

[37] Kahn CR Wang G Lee KY. Altered adipose tissue and adipocyte function in the pathogenesis of metabolic syndrome. J Clin Invest. (2019) 129(10):3990–4000. doi: 10.1172/JCI129187 31573548 PMC6763230

[38] Jiang L Shi K Guo YK Ren Y Li ZL Xia CC. The additive effects of obesity on myocardial microcirculation in diabetic individuals: A cardiac magnetic resonance first-pass perfusion study. Cardiovasc Diabetol (2020) 19(1):52. doi: 10.1186/s12933-020-01028-1 32375795 PMC7201945

[39] de Mutsert R Sun Q Willett WC Hu FB van Dam RM. Overweight in early adulthood, adult weight change, and risk of type 2 diabetes, cardiovascular diseases, and certain cancers in men: A cohort study. Am J Epidemiol. (2014) 179(11):1353–65. doi: 10.1093/aje/kwu052 PMC4036209 24786797

[40] Jung HS Chang Y Eun Yun K Kim CW Choi ES Kwon MJ. Impact of body mass index, metabolic health and weight change on incident diabetes in a Korean population. Obes (Silver Spring). (2014) 22(8):1880–7. doi: 10.1002/oby.20751 24706434

[41] Messerli FH Hofstetter L Rimoldi SF Rexhaj E Bangalore S. Risk factor variability and cardiovascular outcome: JACC review topic of the week. J Am Coll Cardiol (2019) 73(20):2596–603. doi: 10.1016/j.jacc.2019.02.063 31118154

[42] Johnston CA Moreno JP Foreyt JP. Cardiovascular effects of intensive lifestyle intervention in type 2 diabetes. Curr Atheroscler Rep (2014) 16(12):457. doi: 10.1007/s11883-014-0457-6 25288176 PMC5321176

[43] Gibbs BB Brancati FL Chen H Coday M Jakicic JM Lewis CE. Effect of improved fitness beyond weight loss on cardiovascular risk factors in individuals with type 2 diabetes in the look AHEAD study. Eur J Prev Cardiol (2014) 21(5):608–17. doi: 10.1177/2047487312462823 PMC3812302 23012688

[44] Handelsman Y Bloomgarden ZT Grunberger G Umpierrez G Zimmerman RS Bailey TS. American Association of clinical endocrinologists and American college of endocrinology - clinical practice guidelines for developing a diabetes mellitus comprehensive care plan - 2015. Endocr Pract (2015) 21 Suppl 1(Suppl 1):1–87. doi: 10.4158/EP15672.GL 25869408 PMC4959114

[45] Chinese Diabetes Society. Guidelines for the prevention and treatment of T2DM in China. Chin J Diabetes Mellitus (2021) 13(4):315–409. doi: 10.3760/cma.j.cn115791-20210221-00095

[46] Lau DC. Diabetes and weight management. Prim Care Diabetes. (2010) 4 Suppl 1:S24–30. doi: 10.1016/S1751-9918(10)60006-X 20394888

[47] Wong MCS Wang HHX Kwan MWM Fong BCY Chan WM Zhang DX. Dietary counselling has no effect on cardiovascular risk factors among Chinese grade 1 hypertensive patients: A randomized controlled trial. Eur Heart J (2015) 36(38):2598–607. doi: 10.1093/eurheartj/ehv329 26264550

[48] Hu XJ Wu HF Li YT Wang Y Cheng H Wang JJ. Influence of health education on clinical parameters in type 2 diabetic subjects with and without hypertension: A longitudinal, comparative analysis in routine primary care settings. Diabetes Res Clin Pract (2020) 170:108539. doi: 10.1016/j.diabres.2020.108539 33161048

[49] Niswender K. Diabetes and obesity: Therapeutic targeting and risk reduction - a complex interplay. Diabetes Obes Metab (2010) 12:267–87. doi: 10.1111/j.1463-1326.2009.01175.x 20380648

[50] McAdam-Marx C Mukherjee J Bellows BK Unni S Ye X Iloeje U. Evaluation of the relationship between weight change and glycemic control after initiation of antidiabetic therapy in patients with type 2 diabetes using electronic medical record data. Diabetes Res Clin Pract (2014) 103:402–11. doi: 10.1016/j.diabres.2013.12.038 24503045

[51] Wang HHX Li YT Wong MCS. Leveraging the power of health communication: Messaging matters not only in clinical practice but also in public health. Hong Kong Med J (2022) 28(2):103–5. doi: 10.12809/hkmj215128 35470802

[52] Soleymani T Daniel S Garvey WT. Weight maintenance: Challenges, tools and strategies for primary care physicians. Obes Rev (2016) 17(1):81–93. doi: 10.1111/obr.12322 PMC4715703 26490059

[53] Wang HHX Li YT Wong MCS. Strengthening attributes of primary care to improve patients’ experiences and population health: From rural village clinics to urban health centres. Hong Kong Med J (2022) 28(4):282–4. doi: 10.12809/hkmj215133 35989432

[54] Semlitsch T Stigler FL Jeitler K Horvath K Siebenhofer A. Management of overweight and obesity in primary care-a systematic overview of international evidence-based guidelines. Obes Rev (2019) 20(9):1218–30. doi: 10.1111/obr.12889 PMC6852048 31286668

[55] Anderson J Kushner R Miller E Nadglowski J Still C. Overweight and obesity management for primary care clinicians: Executive summary. Clin Diabetes. (2022) 41(1):85–9. doi: 10.2337/cd22-0082 PMC9862448 36714253

[56] Swift DL McGee JE Earnest CP Carlisle E Nygard M Johannsen NM. The effects of exercise and physical activity on weight loss and maintenance. Prog Cardiovasc Dis (2018) 61:206–13. doi: 10.1016/j.pcad.2018.07.014 30003901

[57] Kim JY. Optimal diet strategies for weight loss and weight loss maintenance. J Obes Metab Syndr (2021) 30:20–31. doi: 10.7570/jomes20065 33107442 PMC8017325

[58] Tamura U Tanaka T Okamura T Kadowaki T Yamato H Tanaka H. Changes in weight, cardiovascular risk factors and estimated risk of coronary heart disease following smoking cessation in Japanese male workers: HIPOP-OHP study. J Atheroscler Thromb (2010) 17(1):12–20. doi: 10.5551/jat.1800 20081325

[59] Takayama S Takase H Tanaka T Sugiura T Ohte N Dohi Y. Smoking cessation without educational instruction could promote the development of metabolic syndrome. J Atheroscler Thromb (2018) 25(1):90–7. doi: 10.5551/jat.40063 PMC5770227 28592705

[60] Wannamethee SG Shaper AG Lennon L. Reasons for intentional weight loss, unintentional weight loss, and mortality in older men. Arch Intern Med (2005) 165(9):1035–40. doi: 10.1001/archinte.165.9.1035 15883243

[61] Wang HHX Li YT Zhang Y Wong MCS. Revisiting primary healthcare and looking ahead. Hong Kong Med J (2023). doi: 10.12809/hkmj235139. Epub ahead of print 36740222

